# Leveraging Online Learning to Promote Systems Thinking for Sustainable Food Systems Training in Dietetics Education

**DOI:** 10.3389/fnut.2021.623336

**Published:** 2021-03-18

**Authors:** Marie Spiker, Amanda Hege, Janice Giddens, Joanna Cummings, Jasia Steinmetz, Angie Tagtow, Erin Bergquist, Lauren Burns, Christina Campbell, Diane Stadler, Elizabeth Combs, Nancy Prange, Aaron Schwartz, Katie Brown, Kevin Sauer

**Affiliations:** ^1^Academy of Nutrition and Dietetics Foundation, Chicago, IL, United States; ^2^Nutritional Sciences Program and Department of Epidemiology, University of Washington School of Public Health, Seattle, WA, United States; ^3^Nutrition and Health Care Management, Appalachian State University, Boone, NC, United States; ^4^National Dairy Council, Chicago, IL, United States; ^5^Graduate Programs in Human Nutrition, Oregon Health & Science University, Portland, OR, United States; ^6^School of Health Sciences and Wellness, University of Wisconsin-Stevens Point, Stevens Point, WI, United States; ^7^Äkta Strategies, LLC, Elkhart, IA, United States; ^8^Department of Food Science and Human Nutrition, Iowa State University, Ames, IA, United States; ^9^Department of Dietetics and Human Nutrition, University of Kentucky, Lexington, KY, United States; ^10^School of Health Studies, Northern Illinois University, DeKalb, IL, United States; ^11^Department of Food, Nutrition, Dietetics and Health, Kansas State University, Manhattan, KS, United States

**Keywords:** nutrition, food systems, sustainability, systems thinking, higher education

## Abstract

Educating and training a multisectoral food systems workforce is a critical part of developing sustainable, resilient, and healthy food and water systems. This paper shares perspectives from a working group of educators, learners, and food systems subject matter experts that collaborated over the course of a year to develop, pilot test, and evaluate two interactive webinar series with a multi-site cohort of dietetics interns and graduate students. The three-part webinar series format included a training webinar, a practice activity, and a synthesis webinar. In reflecting on the effectiveness of this format, we provide direct assessments of student learning from subject matter experts alongside indirect assessments from pre- and post-surveys fielded with learners. Learners who participated in an interactive webinar series demonstrated skills in several dimensions of systems thinking and gained confidence in food systems learning outcomes. Learners also shared valuable feedback on the opportunities and challenges of using online platforms for this experience. As online learning opportunities become more common, it will become increasingly important for educators to prioritize strategies that effectively equip students with the higher-order thinking skills, such as systems thinking, needed to address the complexities of sustainable food systems. The interactive webinar series format described here provides an opportunity to leverage didactic webinars in combination with interactive experiences that enable learners to deepen their knowledge through practice with peers and subject matter experts. Though this format was piloted within dietetics education programs, many of the lessons learned are transferable to other food systems educational contexts.

## Introduction: A Growing Need for Effective Online Learning in Sustainable Food Systems

It is widely recognized that food systems are both drivers and outcomes of sustainability and are integral to meeting the Sustainable Development Goals (SDGs) (1, 2). A sustainable food system is one that “delivers food security and nutrition for all in such a way that the economic, social and environmental bases to generate food security and nutrition for future generations are not compromised” (3). A critical part of this work is educating and training a multisectoral food systems workforce. A workforce that can support sustainable food systems is diverse and includes, among others, scientists, health and public health professionals, and stakeholders in public policy, civil society, and the private sector. Though these career pathways may be divergent, higher education is a point of intervention for training stakeholders to support the shared goals of sustainable resilient, and healthy food and water systems.

In training a sustainable food systems workforce, systems thinking is a skillset that is increasingly valued (4). In this work, we draw from two existing systems thinking definitions. Arnold and Wade (5) define systems thinking as “a set of synergistic analytic skills used to improve the capability of identifying and understanding systems, predicting their behaviors, and devising modifications to them in order to produce desired effects.” Valley et al. (6) center systems thinking on the principles of holism and pluralism, where holism refers to perceiving the interactions between system components and pluralism refers to “the explicit engagement and valuing of multiple perspectives in defining systems objectives, boundaries, interventions, and evaluations.” Drawing from these definitions, we frame systems thinking as an analytical skillset that engages multiple perspectives, including those from multiple scientific disciplines and multiple sectors beyond scientific research.

This Perspectives article describes insights from an interdisciplinary working group on the development and pilot testing of an interactive webinar series format designed to facilitate the development of systems thinking skills in a multi-site cohort of dietetics interns and graduate students. Most learners at this stage have completed an undergraduate degree with an emphasis in nutrition and dietetics and are pursuing a credential as a registered dietitian nutritionist through a dietetic internship or graduate program.

Nutrition and dietetics professionals are important food systems change agents: they influence individual food choices through direct interaction with patients and clients, and these choices have rippling effects throughout the food system. In their varied roles throughout food systems, these professionals also influence policy, system, and environmental change (7). Thought leaders in this profession have recognized the importance of food systems (8–12, 14), with some further emphasizing the multi-dimensionality of sustainable food systems as including environmental stewardship; economic vitality; and social, cultural, and ethical capital (13, 15, 16). Carino et al. (17) highlighted that this multi-dimensionality requires dietetics education programs to prioritize higher-order learning outcomes and assessments.

Though this paper provides insights developed within the context of dietetics education, these perspectives are transferable across disciplines and sectors. Food systems is growing as a program of study: the first interdisciplinary food studies program in the United States began in the 1990's, and as of 2015 Hartle and colleagues identified 22 doctoral, 36 masters- or graduate certificate-level, and 82 undergraduate food systems programs in the United States (18). Within nutrition and dietetics, educational programs are expanding offerings related to food systems (19–21). Within nutrition more broadly, including public health nutrition, Shrimpton et al. (22) and Fanzo et al. (23) described the need to build global nutrition capacity for the multisectoral collaborations needed to meet the SDGs. Within allied health professions, efforts are underway to incorporate cross-cutting principles of planetary health education (24) into interprofessional clinical education in nursing (25–28) and related professions (29). Public health education is also incorporating sustainable food systems content through both curricula and competencies (30).

Amidst an expansion of food systems educational programs and the rapid proliferation of online learning, educators will need to prioritize options that effectively equip learners with the higher-order thinking skills needed for multisectoral food systems work. The objective of this paper is to share perspectives on how food systems educators can use online learning to facilitate the development of systems thinking for future food systems professionals. In this paper we use the term online learning to refer broadly to pedagogical methods that use online tools for communication and collaboration, recognizing the existence of similar and evolving terminology (31).

## Methods: Developing the Interactive Webinar Series Format

**Figure 1:**
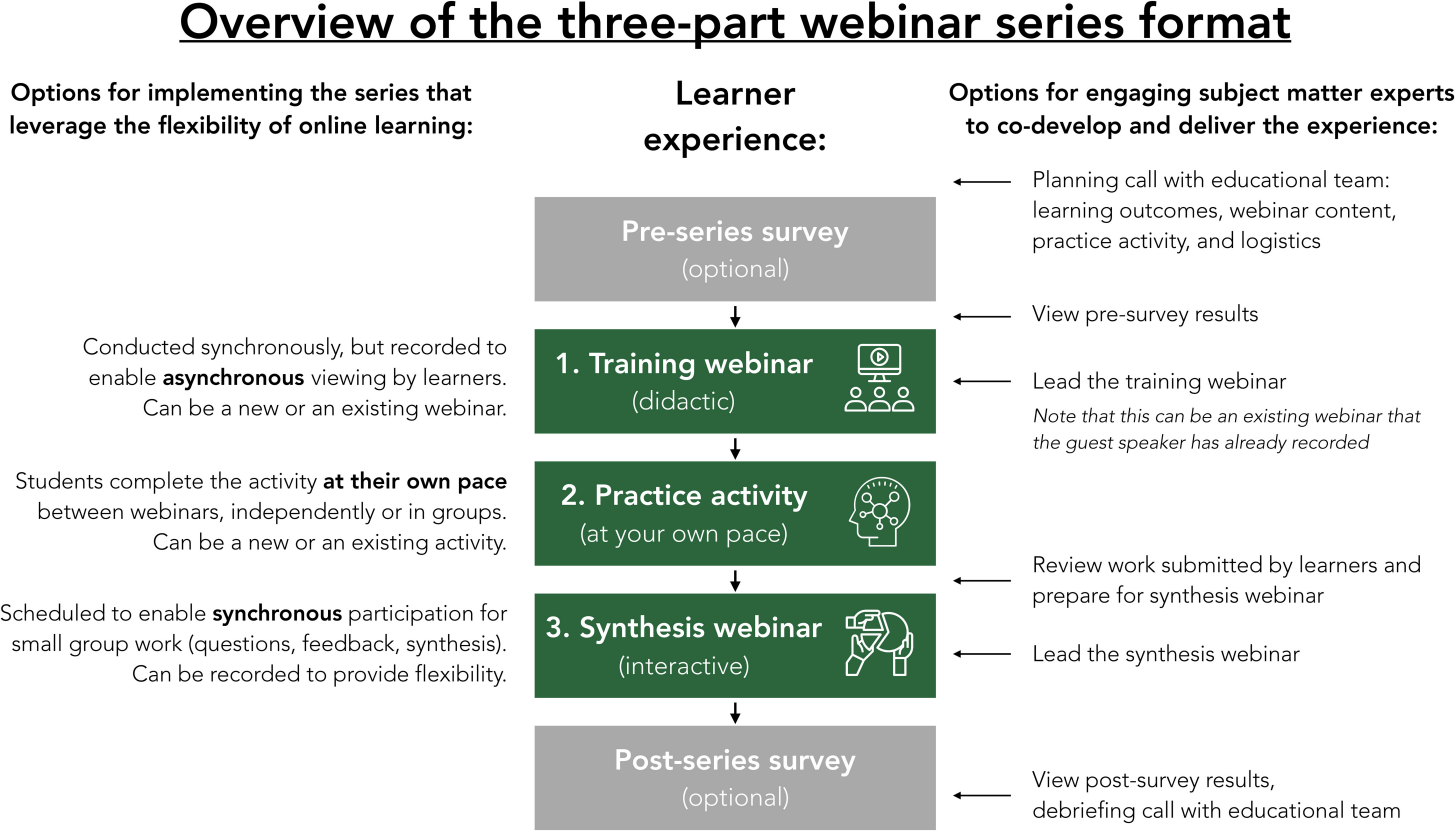
The three-part webinar series format included a training webinar, a practice activity, and synthesis webinar. In developing and piloting the two series described in this paper, we used pre- and post- surveys to assess effectiveness and increase opportunities for engagement with subject matter experts. This figure describes ways the format can be implemented to leverage the flexibility of online learning, as well as possible points of contact with subject matter experts. This format is dynamic as educators and learners continue to adapt.

This paper provides insights from a working group that collaborated over the course of a year. The working group developed and pilot tested two interactive webinar series following the format shown in Figure 1. The three-part format included a training webinar, a practice activity, and a synthesis webinar. All activities were conducted online between March and May, 2019. Materials from these sessions are available online including training webinar recordings, practice activity templates, and a discussion guide (32). Both series, while focusing on different topical areas within nutrition, introduced opportunities to practice systems thinking and consider connections between students' core training in dietetics and the challenges of sustainable food systems.

The working group was convened by the Academy of Nutrition and Dietetics Foundation, which released a Sustainable, Resilient, and Healthy Food and Water Systems Curriculum for dietetics students and interns (21) as part of its Future of Food Initiative, which was supported by an educational grant from National Dairy Council (32). The 20-person working group included stakeholders from the Future of Food Initiative, directors of dietetics education programs (henceforth: educators), and dietetics interns and graduate students (henceforth: learners) from four university sites implementing the curriculum in the United States. The four university sites included dietetics internship programs and coordinated graduate programs at Iowa State University, Oregon Health & Science University, Northern Illinois University, and the University of Kentucky. The programs collectively enrolled over 140 learners at any given time through in-person, distance, and hybrid programs. Because the series were offered as optional activities beyond the required curricula at each site, and because the four programs operated on independent timelines, the two series were not attended by identical groups of students.

Based on topics prioritized by the working group, subject matter experts serving as guest speakers were identified and participated in the co-development of each series, including designing an activity that would enable learners to practice systems thinking skills between webinars. For the first series, titled “Gaining Ground: Applying Individual, Policy, System, and Environmental (I+PSE) Change to Sustainable Food System Initiatives,” the practice activity guided learners to brainstorm examples within each stage of the I+PSE Conceptual Framework for Action for a specific food systems issue. The I+PSE Conceptual Framework describes a continuum of strategies, ranging from individual to systems change, that individual practitioners can leverage to address adaptive challenges. For the second series, titled “Exploring Malnutrition Through the Lens of Systems Thinking,” the practice activity was an impact analysis where learners considered primary, secondary, and tertiary impacts of nutrition interventions in five domains (ecological, agricultural, economic, socio-cultural, or health impacts). Drawing from the work of Grohs et al. (33), learners were presented with case studies that provided an opportunity to integrate knowledge from multiple domains of sustainable food systems, identify impacts with increasing complexity, and consider stakeholders involved with each impact.

Whereas the training webinars were didactic in nature, the synthesis webinars were interactive, by way of a videoconferencing platform that enabled breakout rooms and online slide templates that enabled collaborative editing. During the didactic webinar, learners were assigned a specific focus for the practice activity. During the synthesis webinar, learners from each focus were placed in smaller groups for discussion before returning to the larger group for feedback and synthesis with subject matter experts, a method known as the “jigsaw” technique (34). During breakout rooms, learners visually shared and refined their ideas using collaborative slides that were pre-populated with templates specific to each series. The breakout room functionality allowed subject matter experts to circulate among small groups, in the same way an instructor might physically circulate around a classroom.

In this Perspectives article we provide insights from an interdisciplinary group of educators and food systems experts on the successes and challenges of teaching and assessing systems thinking skills with dietetics students in an online environment, complemented by learners' assessments of their self-efficacy with systems thinking skills from pre- and post-surveys. The survey instruments appear in Appendix A (Supplementary Material). The instruments and protocols were reviewed and classified as exempt by the University of Kentucky Institutional Review Board (protocol #46256).

## Results: Working Group Perspectives and Learner Feedback From Piloting the Interactive Webinar Series Format

### Insights From Subject Matter Experts and Educators

The development and implementation of these learning opportunities connected a network of 20 educators, learners, and subject matter experts representing eight different institutions, and most working group members are part of the co-author team. Here, this group offers insights on how this experience might inform future efforts to leverage field-specific training such as dietetics within larger collaborative efforts to support sustainable food systems.

In the first series–individual, policy, system, and environmental change–the didactic webinar introduced the I+PSE Conceptual Framework and reviewed a case study of a city ordinance that permitted homeowners to keep chickens and bees in their backyard. In the practice activity, learners brainstormed examples within each component of the framework for a specific food systems issue such as school gardening curriculum, mobile processing, or residential composting. In reviewing the submitted activities, subject matter experts assessed whether students were able to provide examples for each component of the framework and whether the examples accurately reflected each component (e.g., did examples of “changing organizational practice” accurately reflect this concept?). Reflecting on the submitted practice activities and the synthesis webinar, learners generated the most accurate examples of how to change organizational practice, closely followed by suggestions for how to modify physical space. In terms of the number of and accuracy of examples generated, learners had the most difficulty identifying policy and legislation examples.

In the second series–malnutrition through the lens of systems thinking–the didactic webinar introduced the impact analysis method and presented two case studies of malnutrition. Learners completed practice activities where they conducted an impact analysis for two distinct areas of impact. In reviewing the submitted activities, subject matter experts assessed whether students were able to generate a logical flow of primary, secondary, and tertiary impacts, and whether those impacts were aligned with the impact area (e.g., were the “ecological” impacts within the ecological domain?). Learners submitted activities based on a case study of malnutrition in a high-income or lower-middle income country setting; the activities for these two case studies were completed with 88 and 75% accuracy, respectively, as assessed by the subject matter expert. During the synthesis webinar discussions, learners were able to explain a logical thought process in their analysis which was realistic for the case studies given. They demonstrated systems thinking skills by identifying stakeholders, beyond direct recipients of nutrition interventions, who would be affected by a nutrition intervention. Social impacts of nutrition interventions were more challenging for learners. Though learners in both case study groups were able to recognize “increased social stigma” that may accompany certain food recommendations, they were less able to analyze tertiary level impacts of changing relationships around food choice.

Across the two series, learners demonstrated skills in several of Arnold and Wade's dimensions of systems thinking including recognizing interconnections between system components, understanding systems at different scales, and using conceptual models to represent complexity (5). For example, learners recognized that dietitians may increasingly partner with actors such as community non-profit organizations, grocers, and environmentalists in developing a healthier, more sustainable food system. Aligned with Valley et al.'s (6) emphasis on the importance of pluralism to systems thinking, learners also engaged with multiple perspectives. Although it was challenging for learners to draw from disciplinary knowledge outside their primary area of training, the practice activities facilitated student learning in this area by encouraging them to think about how nutrition interventions might play out in multiple areas of impact.

### Insights From Learners

**Figure 2:**
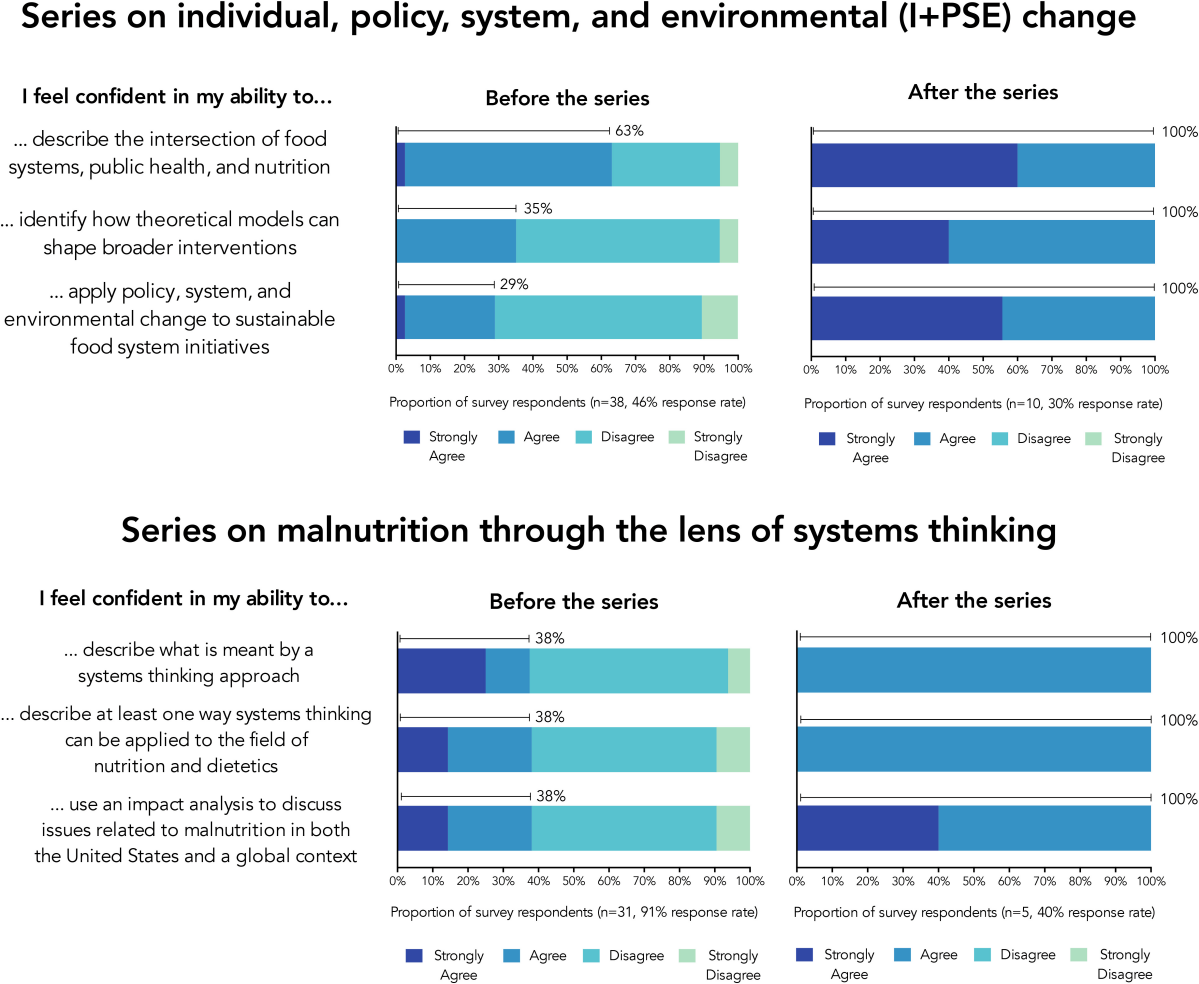
Survey results before and after participation in interactive webinar series. The values above the bars show the sum of “strongly agree” and “agree” responses. In addition to participants' self-reported confidence in learning outcomes (shown here), the surveys also asked about what participants hoped to learn (pre-survey) and ideas for improving the series (post-survey); full survey instruments are available in Appendix A (Supplementary Material), and numerical values for the proportions shown in this figure are available in Appendix B (Supplementary Material).

Across both webinar series, the pre-test results demonstrated a range of self-reported confidence in the learning outcomes, and the post-test results demonstrated more consistent confidence in the learning outcomes, as shown in Figure 2. In the first series–individual, policy, system, and environmental change−83 learners attended the training webinar, 21 learners submitted a practice activity, and 30 learners attended the synthesis webinar. In the pre-survey (*n*=38, 46% response rate), 29–63% of respondents (*n*=11 to 24) agreed or strongly agreed that they had confidence in their ability to perform the learning outcomes. In the post-survey (*n*=10, 30% response rate), all respondents (100%, *n*=9 to 10) expressed confidence in their ability to perform the learning outcomes. In the second series–malnutrition through the lens of systems thinking−34 learners attended the training webinar, 10 learners submitted a practice activity, and 12 learners attended the synthesis webinar. In the pre-survey, (*n*=28, 91% response rate), 38% of respondents (*n*=6 to 9) agreed or strongly agreed that they had confidence in their ability to perform the learning outcomes. In the post-survey (*n*=5, 40% response rate) all respondents (100%, *n*=4 to 5) expressed confidence in their ability to perform the learning outcomes.

In open-ended survey questions, respondents reported that doing the practice activity and interacting with peers and subject matter experts were particularly helpful. The interactive nature of the synthesis webinar helped learners to process more complex topics. One respondent reported, “I do not learn well from [diagrams] and have a hard time understanding them.” Opportunities for interaction supported the ability of learners to progress through the levels of Bloom's taxonomy and achieve greater confidence in the learning outcomes. Learners also reported they found it particularly worthwhile to interact with subject matter experts and peers from other institutions. During the synthesis webinar, the breakout room compositions were designed to facilitate connections between university sites. One learner reported that without exposure to the subject matter experts, who were nutrition professionals working in sustainable food systems, many students would lack the opportunity to know that practitioners in their field can engage in this type of interdisciplinary work.

Respondents also shared opportunities for improvement. Respondents recommended ensuring there were no audiovisual or technical issues during the synthesis webinar, which relied on the functionality of the “breakout room” video-conferencing option; scheduling the webinars more closely together (one or two weeks, instead of three); allowing learners to choose their own topics for the practice activity; and allowing learners to exchange contact information to communicate and work on the practice activity collectively.

Respondents commented on both opportunities and challenges of the jigsaw technique specifically. Learners reported that they liked working through a particular example, they appreciated the opportunity to hear from others, and they appreciated seeing the full breadth of examples shared by the larger group (“it helped me brainstorm ideas for my topic as well”). One respondent noted that even though they were not able to complete the practice activity, “listening to the responses from the other students … was helpful in getting us to think about possible initiatives and changes.” A challenge of the jigsaw technique is that it requires a critical mass of learners to complete preparatory work. Some respondents noted that their small group discussions were less productive because fewer people were prepared to engage in discussion, but they noted that reconvening in the larger group ensured there were still opportunities to learn.

## Discussion: Broader Insights on Transdisciplinary Education in Sustainable Food Systems

### A Wide Network of Collaborators Can Open New Doors for Educators and Learners

As leaders in higher education seek to build capacity for multisectoral collaboration in food systems, this work requires a wider network of collaborators. Educators housed within a specific institutional division may find themselves searching for collaborators in another department, school, university, or a sector outside of academia such as agriculture, urban planning, or government. The two interactive webinar series piloted here represented collaborative efforts between experts in public health nutrition, clinical dietetics, government, agriculture, and systems science. As a result, learners in four dietetics programs were able to benefit from programming that might not have emerged at a single site.

Food systems careers increasingly involve complex problem solving that requires expertise in systems thinking, interdisciplinarity, and intra-professional and inter-professional communication (4, 35). These interactive experiences facilitated connections between learners and subject matter experts outside of their home institutions. During the synthesis webinars, subject matter experts assessed that learners were able to recognize other stakeholders impacted by decisions made by nutrition professionals. These other stakeholders included farmers, climate scientists, and community leaders. Discussions centered around learners and practitioners recognizing that their work as nutrition professionals has wider impacts and that collaboration is needed for systems thinking to guide decisions made within any particular profession. The interconnected nature of global food systems challenges requires a community of practice that is increasingly inclusive and collaborative (36).

### Online Learning Presents Both New Opportunities and Challenges

Innovative technologies have increased the ubiquity and effectiveness of online learning. In addition to the expansion of online learning necessitated by the coronavirus pandemic, several secular trends underlie this shift, including a growing acceptance of remote and hybrid workplaces. Additionally, because transdisciplinary training in food systems may be sought by mid-career professionals who are adding food systems training to an existing set of skills from another field such as policy, public health, social work, or dietetics, learners will include non-traditional students for whom flexible, online training is required. There are also geographic opportunities; food systems transcend geopolitical borders, and remote collaboration helps educators to incorporate perspectives that might otherwise be missing. In addition to researchers who hold formalized knowledge, stakeholders such as food producers, policymakers, and culinary professionals possess embodied knowledge of food systems (37). Online platforms offer a way to tap into this expertise with judicious use of time and financial resources. Developing methods for systematically engaging experts from multiple countries, disciplines, and sectors can expand the accessibility and inclusivity of this training to a wider variety of programs and learners.

As online learning opportunities proliferate, it will become increasingly important for educators to prioritize strategies that effectively equip learners with the higher-order thinking skills needed to address the complexities of sustainable food systems. Whereas lecture-style webinars typically emphasize the lower levels of Bloom's taxonomy (remember and understand), the practice activity and synthesis webinar enabled learners to practice applying, analyzing, evaluating, and creating (38). In this pilot, the format of small group analysis and discussion mimics conversations taking place at all levels of society where stakeholders are becoming more cognizant of both positive and negative impacts of professional practice on sustainability. The interactive webinar series described in this paper represent one possible format for dialogues that contribute toward achieving the SDGs.

Online learning requires additional attention to ensure that the technology employed–including any videoconferencing and collaboration tools–serve to enhance rather than detract. Some tools for online learning can approximate or enhance in-person experiences; for example, videoconferencing breakout rooms can enable learners to share their collaborative work with instructors in real-time, facilitating frequent formative assessments (39). However, online learning can also detract from the experience when technology is not consistently functional or when learners do not have equitable access to the hardware, software, internet connectivity, or physical workspaces needed to fully optimize the use of these tools.

### Strengths, Limitations, and Future Research

A strength of this work is that it provides insights from an instructional method that engages a diverse group of multisectoral food systems stakeholders while traversing challenges such as working across different universities, sectors, and geographic settings. The flexibility of this method enables instructors to integrate new educational content while educational programs are underway and while using time and financial resources efficiently.

This paper includes results from pre- and post-surveys. Limitations of these surveys include variable response rates (ranging from 30–91% of participants) and relatively small sample sizes (ranging from 5 to 38 respondents per survey), which are due to these opportunities having been offered as optional activities for students in four separate dietetics programs. For most sites the development and implementation of these opportunities occurred midway through their program cycles rather than being incorporated as requirements from the start, which contributed to the variable response rates and attrition within each series. However, these circumstances also reflect the dynamic and iterative way that dietetics educators update their programs from one year to the next. Another limitation of this work is that the pre- and post-surveys assessed students' confidence in the learning outcomes, which is an indirect assessment of the construct of self-efficacy (40, 41) rather than a direct assessment of students' knowledge of systems thinking. However, this indirect assessment was complemented by direct assessments of student learning from the subject matter experts who developed and implemented each series.

Arnold and Wade (5) emphasize the difficulty of creating an operational definition of systems thinking; related to this, educators have the challenge of assessing the extent to which students possess systems thinking skills. It is challenging to capture direct assessments of more complex skills in pre- and post- assessments that have a low burden of participation and evaluation. In this pilot, we prioritized a low burden of participation and evaluation because the two series were offered to students as optional activities and were co-developed by subject matters experts on a voluntary basis. This reflects a growing demand for interdisciplinary food systems education alongside a relatively limited capacity to meet this demand within dietetics education programs. Incorporating systems thinking as a core element within programs would provide educators an opportunity to assess this complex skill more comprehensively at the program level. We hope to see a proliferation of further pedagogical research on methods for assessing performance in systems thinking and other higher-order skills that are needed for collaborative, multisectoral work in sustainable food systems.

## Conclusions

This paper provides insights from a working group that collaborated to develop, pilot test, and evaluate an interactive webinar series format to introduce complex food systems topics to dietetics interns and graduate students. These perspectives are transferable to other fields of study within sustainable food systems more broadly, and we hope these insights can provide educators and learners a means to navigate an evolving field that continues to challenge disciplinary and sectoral boundaries.

Educators may find that online learning provides flexibility to engage experts with both formal and embodied knowledge of food systems practices, policies, and outcomes. While a single webinar can be a useful learning tool, the interactive webinar series format described here provides an opportunity to leverage lecture-style or didactic materials in combination with interactive activities that are more conducive to teaching higher-order thinking skills such as systems thinking. We encourage educators to build on existing resources, expand their collaborative networks across the food system, partner with educational programs at other sites, and leverage online tools that provide flexibility for all parties.

Learners pursuing careers in sustainable food systems may find that building connections with peers and food systems stakeholders is critical to navigate an area where training and career pathways are evolving. Opportunities such as those described here can prepare learners for workplaces that are increasingly requiring proficiency with remote communication and transdisciplinary collaboration.

This work also has implications for a growing food systems workforce that includes stakeholders in public policy, civil society, and the private sector. By convening food systems stakeholders with students in educational spaces online, higher education programs can facilitate the exchange of ideas across sectors. In addition to teaching students about food systems, educators can contribute to positive change within these very systems.

## Data Availability Statement

The original contributions presented in the study are included in the article/Supplementary Material, further inquiries can be directed to the corresponding author/s.

## Author Contributions

EB, KB, LB, CC, EC, JG, AH, NP, AS, KS, MS, and DS were members of the working group that conceptualized and evaluated the educational opportunities described in this paper. JC, JS, and AT were subject matter experts who co-developed and implemented the educational opportunities. MS wrote the first draft of the manuscript. All authors provided critical revisions on manuscript drafts and approved the final manuscript.

## Conflict of Interest

MS performed this work as part of an Academy of Nutrition and Dietetics Foundation Fellowship and AH performed this work as part of a contract role with the Academy of Nutrition and Dietetics Foundation; both roles were funded through an educational grant from the National Dairy Council. MS has received travel support for speaking engagements from the American Frozen Food Institute (2015) and from the Academy of Nutrition and Dietetics Foundation through an educational grant from Bayer Crop Science (2019), and honoraria from the Ohio State University. KB and JG performed this work as part of their employment at National Dairy Council. KS supported this work partly as a liaison appointed by the Academy of Nutrition and Dietetics, and partly through professional experience with the subject matter; no compensation occurred as a result of this work. AT receives consultancies from UCLA Fielding School of Public Health, Union of Concerned Scientists, The Healthiest State Initiative, Association of State Public Health Nutritionists, and the Kellogg Fellows Leadership Alliance. AT has received honoraria from the University of Mississippi, Duke University, Green Mountain College, Iowa State University Leopold Center for Sustainable Agriculture, Kansas Nutrition Council, Iowa Department of Public Health, and the Montana Academy of Nutrition and Dietetics. AT serves as a founding Board member to Feed the Truth which is funded by the Lubetzky Family Foundation. AT served as a political appointee/Senior Executive Service and Executive Director for the USDA Center for Nutrition Policy and Promotion from 2014 to 2017. The remaining authors declare that the research was conducted in the absence of any commercial or financial relationships that could be construed as a potential conflict of interest.
