# Supplementary material for: Leveraging Online Learning to Promote Systems Thinking for Sustainable Food Systems Training in Dietetics Education
Source: Front Nutr. 2021 Mar 18;8:623336. doi: 10.3389/fnut.2021.623336 (PMC8012755; doi:10.3389/fnut.2021.623336)
Supplement: Supplementary file 1 [file Data_Sheet_1.pdf]

Supplemental Material for:

Spiker M, Hege A, Giddens J, Cummings J, Steinmetz J, Tagtow A, Bergquist E, Burns L, Campbell C, Stadler D, Combs E, Prange N, Schwartz A, Brown K, and Sauer, K (2021), Leveraging Online Learning to Promote Systems Thinking for Sustainable Food Systems Training in Dietetics Education. *Frontiers in Nutrition.*

APPENDIX A: Survey instruments

1. Pre-survey

	Question	Response Option
Q1	Rate the level you agree or disagree with the following statements. I feel confident in my ability to:<br><br>[Insert specific learning outcomes]	SELECT ONE<br><br>() Strongly agree<br>() Agree<br>() Disagree<br>() Strongly disagree
Q2	By the time I complete this activity, I hope to be able to:	OPEN-ENDED (TEXT BOX)
Q3	What else should we know?	OPEN-ENDED (TEXT BOX)

2. Post-survey

	Question	Response Option
Q1	Please indicate which of the following activities you participated in:	CHECK ALL THAT APPLY<br><br>[ ] Attended training webinar (live)<br>[ ] Attended training webinar (watched the recording after)<br>[ ] Worked on the practice activity<br>[ ] Submitted the practice activity<br>[ ] Attended the synthesis webinar (live)<br>[ ] Attended the synthesis webinar (watched the recording after)

Q2	Rate the level you agree or disagree with the following statements. I feel confident in my ability to:<br><br>[Insert specific learning outcomes]	SELECT ONE<br><br>() Strongly agree<br>() Agree<br>() Disagree<br>() Strongly disagree
Q3	The overall activity was:	SELECT ONE<br><br>() Excellent<br>() Good<br>() Satisfactory<br>() Weak<br>() Poor
Q4	What were the most useful aspects of this activity?	OPEN-ENDED (TEXT BOX)
Q5	What would improve this activity for future learners?	OPEN-ENDED (TEXT BOX)
Q6	The overall effectiveness of the facilitator was:	SELECT ONE<br><br>() Excellent<br>() Good<br>() Satisfactory<br>() Weak<br>() Poor
Q7	Please share any additional feedback you may have about the facilitator.	OPEN-ENDED (TEXT BOX)
Q8	Are there any changes to the format that could improve the learning experience for future participants?	OPEN-ENDED (TEXT BOX)
Q9	What else should we know?	OPEN-ENDED (TEXT BOX)

APPENDIX B: Numerical values for survey results shown in Figure 2

Series on individual, policy, system, and environmental (I+PSE) change

	Strongly Agree	Agree	Disagree	Strongly Disagree
**Before the series:** I feel confident in my ability to…				
… describe the intersection of food systems, public health, and nutrition (n, % of 38 respondents)	1 (2.6%)	23 (60.5%)	12 (31.6%)	2 (5.3%)
… identify how theoretical models can shape broader interventions (n, % of 37 respondents)	0 (0%)	13 (35.1%)	22 (59.5%)	2 (5.4%)
… apply policy, system, and environmental change to sustainable food system initiatives (n, % of 38 respondents)	1 (2.6%)	10 (26.3%)	23 (60.5%)	4 (10.5%)
**After the series:** I feel confident in my ability to…				
… describe the intersection of food systems, public health, and nutrition (n, % of 10 respondents)	6 (60%)	4 (40%)	0 (0%)	0 (0%)
… identify how theoretical models can shape broader interventions (n, % of 10 respondents)	4 (40%)	6 (60%)	0 (0%)	0 (0%)
… apply policy, system, and environmental change to sustainable food system initiatives (n, % of 9 respondents)	5 (55.6%)	4 (44.4%)	0 (0%)	0 (0%)

Note that the number of respondents differs by question because survey questions were optional.

Series on malnutrition through the lens of systems thinking

	Strongly Agree	Agree	Disagree	Strongly Disagree
**Before the series:** I feel confident in my ability to…				
… describe what is means by a systems thinking approach (n, % of 16 respondents)	4 (25%)	2 (12.5%)	9 (56.3%)	1 (6.3%)
… describe at least one way systems thinking can be applied to the field of nutrition and dietetics (n, % of 21 respondents)	3 (14.3%)	5 (23.8%)	11 (52.4%)	2 (9.5%)
… use an impact analysis to discuss issues related to malnutrition in both the United States and a global context (n, % of 28 respondents)	1 (3.6%)	8 (28.6%)	15 (53.6%)	4 (14.3%)
**After the series:** I feel confident in my ability to…				
… describe what is means by a systems thinking approach (n, % of 4 respondents)	0 (0%)	4 (100%)	0 (0%)	0 (0%)
… describe at least one way systems thinking can be applied to the field of nutrition and dietetics (n, % of 4 respondents)	0 (0%)	4 (100%)	0 (0%)	0 (0%)
… use an impact analysis to discuss issues related to malnutrition in both the United States and a global context (n, % of 5 respondents)	2 (40%)	3 (60%)	0 (0%)	0 (0%)

Note that the number of respondents differs by question because survey questions were optional.
